# Review on the significance and clinical application of anti-p53 autoantibody detection

**DOI:** 10.3389/fimmu.2026.1910252

**Published:** 2026-10-06

**Authors:** Fan Bu, Zhe-Nan Ling, Pei Li, Bin Zhou, Wang-Yu Zhu, Jiu-Li Wang, Xiang Liu, Tian-Yu Zhu, Yi-An Du, Qiu-Lei Lang, Liu-Hui Zhou, Zi-Yan Jin, Wen-Jing Wang, Yi-Xuan Li, Yu-Zhen Zhang, Xia-Ying Du, Zhi-Qiang Ling

**Affiliations:** 1 Zhejiang Cancer Institute, Zhejiang Cancer Hospital, Hangzhou, Zhejiang, China; 2 Division of Hepatobiliary and Pancreatic Surgery, Department of Surgery, The First Affiliated Hospital, Zhejiang University School of Medicine, Hangzhou, China; 3 Department of Pathophysiology, School of Basic Medical Sciences, Zhengzhou University, High-Tech Zone, Zhengzhou, Henan, China; 4 Department of General Surgery, Taixing People’s Hospital, Yangzhou University, Taixing, Jiangsu, China; 5 Cell and Molecular Biology Laboratory, Zhoushan Hospital, Zhoushan, Zhejiang, China; 6 Department of Medical Oncology, Bozhou Hospital of Traditional Chinese Medicine, Bozhou, Anhui, China; 7 Department of Laboratory Medicine, Tongde Hospital of Zhejiang Province, Hangzhou, Zhejiang, China; 8 Department of Surgery, Jinhua People’s Hospital, Jindong District, Jinhua, Zhejiang, China; 9 Department of Surgery, Zhejiang Hospital, Hangzhou, Zhejiang, China; 10 Department of Molecular Diagnostics, Hangzhou Lianchuan Biotechnology Co., Hangzhou, Zhejiang, China

**Keywords:** cancer diagnosis, immunoassay, P53 autoantibody, precision medicine, tumor marker

## Abstract

As a critical tumor suppressor gene, mutations in p53 often contribute to the initiation and progression of cancer. Anti-p53 autoantibody (p53-AAb), produced by the immune system in response to mutated p53 protein, can be detected in the blood during early stages of tumor development. As a non-invasive biomarker, p53-AAb demonstrates potential for early screening, auxiliary diagnosis, prognosis evaluation, and treatment monitoring across various cancer types. Based on current literature and evidence-based medical data, this Review Article provides an overview of the scientific rationale, technical aspects, and clinical applications of p53-AAb testing. It summarizes practical considerations to guide scientific research and clinical practice, with the aim of promoting standardized utilization of this biomarker.

## 1 Introduction

This Review Article aims to provide clinicians, researchers, and laboratory professionals with guidance on p53 autoantibody testing and its clinical applications, with the goal of promoting standardization in this field and optimizing personalized cancer diagnosis and treatment strategies (1, 2). It seeks to support decision-making for early screening, diagnosis, and precision medicine in patient care, while emphasizing that p53-AAb remains an adjunctive biomarker rather than a stand-alone test (20, 21).

Mechanistically, p53 autoantibodies usually arise when mutant or conformationally altered p53 accumulates in tumor cells, increases antigen exposure, and breaks immune tolerance (1, 2). Seropositivity therefore more often reflects tumor-associated antigen burden and host humoral immune recognition than a direct pathogenic effect (3, 6, 21).

This Review Article may be periodically updated based on advancements in relevant research to meet the needs of both experimental studies and clinical practice. Evidence was gathered from databases including PubMed, CNKI, Wanfang, and VIP, as well as official publicly available information from regulatory authorities such as the National Medical Products Administration (NMPA) and the U.S. Food and Drug Administration (FDA) (search cut-off date: June 30, 2025). Selected studies encompass systematic reviews, meta-analyses, cohort studies, and case-control studies published domestically and internationally (20, 21, 24, 25). The evidence was summarized descriptively according to study design, biomarker relevance, and clinical applicability. For methodological transparency, the literature search used combinations of the terms “p53 autoantibody”, “p53 antibody”, “tumor-associated autoantibody”, “TP53”, “cancer”, “screening”, “diagnosis”, “prognosis”, and “treatment monitoring”, together with cancer-specific terms for gastric, colorectal, breast, lung, esophageal, and oral cancer. We included English- or Chinese-language human studies, systematic reviews, meta-analyses, cohort studies, case-control studies, and relevant methodological or regulatory documents addressing p53-AAb detection or clinical application. We excluded duplicate records, non-human studies, conference abstracts without sufficient data, and reports unrelated to p53-AAb testing. Records were screened for relevance by title/abstract and full text, and the evidence was synthesized narratively. For reference, the Oxford Centre for Evidence-Based Medicine (CEBM) 2011 Levels of Evidence table (**Table 1**) is provided below; formal evidence levels were not assigned to individual citations because this Review Article addresses heterogeneous questions and study designs.

**Table 1:** Oxford Centre for Evidence-Based Medicine 2011 Levels of Evidence.

Question	Step 1 (Level 1*)	Step 2 (Level 2*)	Step 3 (Level 3*)	Step 4 (Level 4*)	Step 5 (Level 5)
How common is the problem?	Local and current random sample surveys (or censuses)	Systematic review of surveys that allow matching to local circumstances**	Local non-random sample**	Case-series**	n/a
Is this diagnostic or monitoring test accurate? (Diagnosis)	Systematic review of cross sectional studies with consistently applied reference standard and blinding	Individual cross sectional studies with consistently applied reference standard and blinding	Non-consecutive studies, or studies without consistently applied reference standards**	Case-control studies, or “poor or non-independent reference standard**	Mechanism-based reasoning
What will happen if we do not add a therapy? (Prognosis)	Systematic review of inception cohort studies	Inception cohort studies	Cohort study or control arm of randomized trial*	Case-series or case- control studies, or poor quality prognostic cohort study**	n/a
Does this intervention help? (Treatment Benefits)	Systematic review of randomized trials or *n*-of-1 trials	Randomized trial or observational study with dramatic effect	Non-randomized controlled cohort/follow-up study**	Case-series, case-control studies, or historically controlled studies**	Mechanism-based reasoning
What are the COMMON harms? (Treatment Harms)	Systematic review of randomized trials, systematic review of nested case-control studies, *n*- of-1 trial with the patient you are raising the question about, or observational study with dramatic effect	Individual randomized trial or (exceptionally) observational study with dramatic effect	Non-randomized controlled cohort/follow-up study (post-marketing surveillance) provided there are sufficient numbers to rule out a common harm. (For long-term harms the duration of follow-up must be sufficient.)**	Case-series, case-control, or historically controlled studies**	Mechanism-based reasoning
What are the RARE harms? (Treatment Harms)	Systematic review of randomized trials or *n*-of-1 trial	Randomized trial or (exceptionally) observational study with dramatic effect			
Is this (early detection) test worthwhile? (Screening)	Systematic review of randomized trials	Randomized trial	Non-randomized controlled cohort/follow-up study**	Case-series, case-control, or historically controlled studies**	Mechanism-based reasoning

* Level may be graded down on the basis of study quality, imprecision, indirectness (study PICO does not match questions PICO), because of inconsistency between studies, or because the absolute effect size is very small; Level may be graded up if there is a large or very large effect size.

** As always, a systematic review is generally better than an individual study.

## 2 Overview of p53 autoantibodies

### 2.1 Role of the p53 gene and mutation mechanisms

The p53 gene, often referred to as the “guardian of the genome,” maintains genomic stability by regulating the cell cycle, DNA repair, and apoptosis. However, mutations in the p53 gene can lead to loss of function or gain of oncogenic functions, which are closely associated with the development of various solid tumors, including lung, breast, gastric, and colorectal cancers. Studies have shown that p53 mutations promote tumor cell proliferation, invasion, and immune evasion (1). As a critical tumor suppressor gene, p53 plays vital roles in apoptosis, growth regulation, differentiation, aging, and DNA repair (2). The wild-type p53 gene prevents normal cells from transforming into tumor cells, hence its designation as the “guardian of the genome”.

### 2.2 Discovery and characteristics of p53 autoantibodies

p53-AAb were first identified in 1982 in sera from patients with breast cancer (5). Subsequent studies confirmed their association with mutations and aberrant accumulation of p53 protein (3, 4). Across cancer types, reported seropositivity rates are usually in the range of 15%-40%, whereas positivity is uncommon in healthy populations (3). Notably, p53-AAb can be detected during early stages of carcinogenesis in selected cohorts, underscoring their potential value in early cancer screening (7, 8).

The initial discovery of p53-AAb occurred in the serum of breast cancer patients (5). Subsequent research demonstrated their presence across diverse cancer types, including colorectal, breast, lung, liver cancers and lymphomas (6). In NSCLC and other lung-cancer cohorts, p53-AAb has generally shown limited sensitivity but relatively high specificity, supporting its use only as part of multi-marker or imaging-integrated strategies (13, 20, 29).

p53-AAb represent products of the immune response triggered by abnormal p53 protein expression, predominantly of the IgG class, with minority IgM and occasional IgA responses observed in cancer patients. Critically, several studies have demonstrated seropositivity for p53-AAb in early-stage tumors that remain clinically undetectable. Regele et al. detected p53-AAb across all stages of breast cancer (*in situ*, invasive, and metastatic), suggesting their emergence as an early event in tumorigenesis (7). Trivers et al. reported detectable p53-AAb in serum prior to the manifestation of clinical symptoms in lung cancer patients (8). These collective findings position serum p53-AAb as promising biomarkers for early cancer detection, highlighting the clinical importance of their detection in cancer management.

### 2.3 Clinical significance and future directions

Studies indicate that the production of p53-AAb can occur early in cancer development (7, 8). In breast cancer, p53-AAb has been detected in a subset of patients and is most appropriately interpreted as complementary serological information rather than as an independent screening tool (5, 7, 26). In colorectal cancer, meta-analytic and cohort data suggest high specificity but low-to-moderate sensitivity, supporting an adjunctive role in screening or perioperative baseline interpretation (9, 10, 24). In gastric cancer, p53-AAb positivity is more closely linked to aggressive clinicopathological features and poorer outcomes than to stand-alone screening performance (11, 15, 27). Esophageal cancer studies similarly support a diagnostic or prognostic role for p53-related autoantibody panels, particularly when combined with other tumor-associated autoantibodies (12, 18, 31, 32).

## 3 Detection methods and standardization procedures

### 3.1 Detection technologies

The primary techniques for detecting p53-AAb include enzyme-linked immunosorbent assay (ELISA), Western blot, protein microarray, and more advanced electrochemical sensor technologies (13, 21, 35). The characteristics and applications of each method are summarized below.

#### 3.1.1 Enzyme-linked immunosorbent assay

ELISA is currently the most widely used method for p53-AAb detection because it is relatively simple, scalable, and compatible with serum-based autoantibody measurement (13, 20). A 2023 multicenter study of a lung-cancer autoantibody panel reported diagnostic value for ELISA-based tumor-associated autoantibody detection in the Chinese population (13). However, results may be influenced by experimental conditions, including antigen concentration, blocking reagent, serum dilution, incubation time, and washing stringency (22, 23, 35).

1. Principle.

ELISA relies on the specific binding of labeled enzymes to antigens and antibodies, with signal amplification through enzymatic substrate reactions to quantify antibody concentrations.

2. Advantages.

High throughput makes ELISA suitable for large-scale research and auxiliary clinical screening (13, 20). Recombinant p53 protein-based ELISA has repeatedly shown high specificity but limited sensitivity in colorectal and gastric cancer studies (9, 11, 24). Similar limitations have been reported in breast, lung, and esophageal cancer evidence, so positive results should be interpreted in clinical context (12, 20, 26).

3. Limitations.

Sensitivity is susceptible to variations in experimental conditions such as antigen concentration and blocking agents. Potential false-positive or false-negative results necessitate validation with complementary methods.

#### 3.1.2 Western blot

Western blot detects the binding of p53-AAb to denatured proteins in samples and serves as a reference method for confirmatory testing. However, it is operationally complex and difficult to quantify, making it unsuitable for large-scale screening.

1. Principle.

Proteins are separated via electrophoresis and subsequently detected using antibodies to identify specific proteins or antibodies.

2. Application.

Primarily used for confirmatory studies to analyze antibody binding to denatured p53 protein.

3. Advantages and Limitations.

High specificity, but the technique is labor-intensive, challenging to standardize, and not suitable for high-throughput screening.

#### 3.1.3 Protein microarray technology

Protein microarray enables high-throughput detection of p53-AAb and other autoantibodies, offering advantages in multi-marker analysis (21, 36). Multi-antigen autoantibody panels have been evaluated in lung and colorectal cancer, and combined panels generally improve sensitivity compared with a single autoantibody while preserving useful specificity (20, 24, 29). Breast and esophageal cancer studies also support panel-based interpretation rather than reliance on a single p53-AAb result (25, 32, 36). Its application requires sophisticated instrumentation and bioinformatics support.

1. Principle.

Multiple antigens are immobilized on microarray chips, and antibody binding is detected via fluorescence labeling to achieve high-throughput screening.

2. Advantages.

Enables simultaneous detection of multiple autoantibodies (e.g., p53-AAb, HER2-AAb) and improves early cancer diagnosis accuracy through combined analysis.

3. Clinical Potential.

Integration of protein microarray with artificial intelligence algorithms holds promise for further enhancing sensitivity in early cancer detection.

#### 3.1.4 Practical considerations

The use of recombinant full-length p53 protein as an antigen is recommended to enhance epitope coverage, detection specificity, and inter-assay stability (3, 6, 13). Future developments may incorporate nanomaterials or electrochemical immunosensors to establish detection platforms with higher sensitivity, but these approaches remain exploratory for p53-AAb and require analytical and clinical validation (34, 35). Integration of artificial intelligence algorithms may help interpret multi-marker autoantibody profiles when trained and validated in independent cohorts (21, 32).

### 3.2 Clinical sample processing

The collection, processing, and preservation of clinical samples significantly influence p53-AAb results. Preanalytical variables such as tube type, anticoagulant choice, clotting time, centrifugation, storage temperature, freeze-thaw cycles, hemolysis, icterus, and lipemia can materially affect serum or plasma protein measurements and immunoassay performance (22, 23, 33). To ensure reliability and reproducibility, standardized processing procedures should be implemented for different sample types. The following section details the handling steps and requirements for serum, plasma, and other potential sample types.

#### 3.2.1 Sample collection and processing

Various sample types can be used in ELISA experiments, including serum, plasma, cell culture supernatant, urine, and tissue homogenates, but clinical p53-AAb studies most consistently use serum (3, 13, 20). Pretreatment methods vary depending on the sample type, and appropriate sample pretreatment is the first step to ensure the accuracy of ELISA results (22, 23, 36).

Because ELISA measures soluble antigen-antibody binding signals, samples should be clear and free of precipitates or suspended solids before testing (22, 23). Storage at -80 C is preferable for longer preservation of protein and antibody measurements, and repeated freeze-thaw cycles should be avoided (22). Sodium azide should not be used in HRP-based assays because it inhibits peroxidase activity and may cause false-negative results (22, 23, 33, 35).

1. Serum.

Serum is the most commonly used specimen type for ELISA-based assays and is considered the preferred sample for p53-AAb detection because it avoids anticoagulant-related matrix effects (22, 23). Serum is also the matrix most frequently reported in clinical p53-AAb studies across colorectal, lung, and broader cancer cohorts (3, 9, 13, 20). The preparation of serum samples is relatively straightforward but requires strict standardization to minimize preanalytical variability and ensure detection accuracy (22, 23). Briefly, peripheral blood samples should be collected using clean tubes without anticoagulants to avoid potential interference with antibody-antigen interactions. Following collection, blood samples should be allowed to clot at room temperature for 10 minutes to 2 hours depending on ambient conditions, or alternatively incubated at 4 C overnight to facilitate complete clot formation. The serum fraction is then separated, with gentle tilting of the tube recommended to increase the contact surface area and improve serum release. Subsequently, samples should be centrifuged at 2–8 C for approximately 5 minutes at 5000–6000 rpm to remove residual cells and debris. The resulting supernatant should be carefully transferred and subjected to ELISA analysis immediately whenever possible. For long-term storage, serum samples should be aliquoted and stored at -20 C or preferably -80 C to minimize degradation and avoid repeated freeze-thaw cycles. If visible precipitates are observed after storage, samples should be centrifuged again before analysis to obtain a clear supernatant. Throughout the entire preparation process, careful control of sample handling conditions is essential to prevent preanalytical factors that may affect antibody-binding specificity and compromise assay sensitivity (22, 23, 33, 36).

2. Plasma.

Plasma is another specimen type for ELISA-based assays and is suitable only when the detection platform has been validated for anticoagulated samples (22). The choice of anticoagulant is critical for maintaining assay performance; EDTA or citrate may be acceptable in validated protocols, whereas heparin can interfere with some antibody-antigen and enzyme-based detection systems (14, 22, 23, 35). Following blood collection, samples should be processed promptly to minimize hemolysis and release of intracellular components. Briefly, peripheral blood samples should be collected into anticoagulant-treated tubes, and the samples should be gently mixed within 30 minutes after collection to ensure adequate anticoagulation. Subsequently, plasma should be separated by centrifugation at 2–8 C for approximately 5 minutes at 5000–6000 rpm, and the supernatant should be carefully transferred for downstream analysis. For long-term storage, plasma samples should be aliquoted and stored at -20 C or preferably -80 C to preserve sample integrity and minimize repeated freeze-thaw cycles. During sample preparation and storage, particular attention should be paid to avoiding hemolysis and excessive lipid contamination, as these factors may affect assay specificity and sensitivity (22, 23, 33). If precipitates are observed after storage, samples should be centrifuged again before use to obtain a clear supernatant suitable for ELISA analysis.

When both serum and plasma are technically feasible, serum is recommended as the preferred specimen for routine p53-AAb testing because it avoids anticoagulant-related interference (22, 23). This recommendation is also consistent with published ELISA-based p53-AAb clinical studies, which predominantly use serum (3, 9, 13, 20). Plasma may be accepted only when the assay has been validated for the specific anticoagulant and matrix (22, 23).

3. Sample Processing Steps.

Blood samples should be processed in a timely manner to preserve antibody integrity and minimize preanalytical variability (22, 23). Specifically, samples should be centrifuged within 2 hours after collection at 3000 x g for 10 minutes to achieve effective separation of serum or plasma. Following centrifugation, the separated serum or plasma should be carefully transferred into sterile, low-protein-binding tubes and aliquoted into appropriate volumes to facilitate subsequent analyses and minimize the risk of antibody degradation caused by repeated freeze-thaw cycles (22). An aliquot volume of 100–500 uL per tube is recommended depending on downstream assay requirements (22, 23, 33).

4. Quality Control Measures.

Hemolysis, icterus, and lipemia should be avoided during collection and processing because these matrix interferences can affect optical and immunoassay signals (23, 33). Sample processing equipment should be regularly calibrated to prevent deviations in centrifugation speed or temperature that may compromise sample quality (22).

5. Other Sample Types.

Besides serum and plasma, other biological fluids may also be considered as potential specimens for p53-AAb detection in specific research and clinical settings (21). Cerebrospinal fluid may be applicable for investigations involving selected neurological tumors; however, antibody concentrations in CSF are generally lower than those observed in peripheral blood, which may influence detection sensitivity. Pleural fluid and peritoneal fluid may provide additional diagnostic information in selected metastatic tumor settings, although their utility for p53-AAb detection requires further validation. Urine represents another potential noninvasive specimen source; however, standardized collection, processing, and analytical protocols for urinary p53-AAb detection have not yet been established. Therefore, alternative body fluids should be regarded as exploratory matrices until analytical validation and clinical performance data are available (21, 22, 36).

6. Practical Considerations.

Hemolysis should be avoided during blood sample collection, as ruptured red blood cells release substances with peroxidase activity that may cause nonspecific color development in ELISA, leading to inaccurate results and false positives. Additionally, samples must be protected from bacterial contamination, as bacteria may contain endogenous HRP, which can also compromise detection accuracy (14, 23, 33, 35).

#### 3.2.2 Sample storage and transportation

1. Short-Term Storage.

Serum and plasma samples can be stored at 4 °C for no more than 48 hours. Short-term storage is suitable for rapid testing scenarios, particularly in standardized laboratory settings where immediate analysis is performed (22, 23).

2. Long-Term Storage.

For extended preservation, samples should be stored at -80 C or below, and repeated freeze-thaw cycles must be avoided (22). If -20 C storage is used, the storage duration should be minimized and validated locally because protein and antibody measurements may vary with storage temperature, duration, and freeze-thaw exposure (22, 23, 33).

3. Transportation Requirements.

To preserve sample integrity and ensure reliable detection of p53-AAb, biological specimens must be maintained under continuous cold chain conditions throughout transportation (22, 23). For short-distance transportation, samples should be stored and shipped at 2–8 C to minimize protein degradation and maintain antibody stability. For long-distance transportation or extended shipping durations, dry ice (-80 C) should be used to maintain frozen conditions and prevent loss of antibody activity. Samples should be transported in validated insulated cold boxes or liquid nitrogen containers, depending on the required storage temperature and transportation duration. During shipment, mechanical vibration and excessive agitation should be minimized, as these factors may contribute to hemolysis, cellular disruption, or protein degradation, potentially affecting downstream assay performance (22, 23, 33).

#### 3.2.3 Special handling requirements for different sample types

1. Hemolyzed Samples.

Hemolysis can significantly interfere with p53-AAb detection, particularly by increasing background noise in optical or fluorescence-based assays (23, 33). For hemolyzed samples, re-collection is strongly recommended rather than attempting simple dilution (23, 33).

2. Icteric or Lipemic Samples.

The high bilirubin or lipid content in icteric or lipemic samples may interfere with detection signals. It is advisable to use low-protein-binding tubes or perform low-speed centrifugation (2000 × g, 10 minutes) to reduce the impact of interfering substances (23, 33).

3. Sample Dilution.

Samples with high antibody concentrations may exceed the detection range and require dilution (typically using PBS at ratios from 1:100 to 1:500). The dilution factor should be validated in advance using positive control samples to ensure accuracy within the linear range (35, 36).

#### 3.2.4 Quality control and standardization recommendations

1. Control Samples.

Include both positive controls (samples known to be positive for p53-AAb) and negative controls (serum from healthy individuals) in each assay batch to monitor specificity and sensitivity (13, 35). The use of standardized calibrators or validated internal reference materials is recommended to improve comparability across laboratories (21, 22).

2. Equipment and Consumables.

All sample processing equipment (e.g., centrifuges, freezers, transport containers) must undergo regular calibration, and environmental conditions (e.g., temperature, humidity) should be consistently monitored. Low-protein-binding consumables are advised to minimize antibody loss due to adsorption (22, 23, 35).

3. Personnel Training.

Testing must be performed by adequately trained personnel, with emphasis on critical steps such as centrifugation speed, sample aliquoting, and cold chain management to ensure full compliance with standardized protocols (22, 35).

#### 3.2.5 Scientific basis for sample processing

1. Delayed centrifugation, hemolysis, lipemia, and other preanalytical factors can release interfering substances or alter optical signals, thereby reducing the reliability of antibody detection (22, 23, 33).
2. Significant differences in antibody stability have been observed between samples stored at –20 °C and those stored at –80 °C, with a notably greater decline in antibody titer after six months in samples maintained at –20 °C.
3. Regarding anticoagulant selection, antibody activity in EDTA-anticoagulated samples is slightly lower than in citrate-anticoagulated samples, but remains substantially higher than in heparin-anticoagulated samples.

### 3.3 Standardized procedures

#### 3.3.1 Enzyme-linked immunosorbent assay

1. Materials and Reagents.

The materials and reagents include recombinant p53 protein as the antigen, expressed in bacterial or mammalian cell systems; 5% skim milk or 0.1% gelatin in PBS as the blocking buffer to reduce non-specific binding; patient serum or plasma containing p53-AAb as the primary antibody source; enzyme-labeled anti-human IgG, commonly HRP-conjugated, as the secondary antibody; TMB as the enzyme substrate; and sulfuric acid as the stop solution (13, 35). These steps follow standard indirect ELISA practice for autoantibody measurement and should be optimized according to validated kit protocols and local SOPs (20, 22).

2. Experimental Steps.

The experimental procedure begins with coating, in which recombinant p53 protein is diluted in carbonate buffer and incubated in a 96-well plate overnight at 4 C (13, 35). Blocking is then performed by adding blocking buffer, such as skim milk or BSA, to reduce non-specific binding (35). Diluted patient serum or plasma is added and incubated, followed by repeated washing with PBS or PBS containing Tween-20 to remove unbound antibodies (13, 35). HRP-conjugated anti-human IgG antibody is then added and incubated, after which TMB substrate is used for color development and absorbance is measured at 450 nm using a microplate reader (13, 35). The exact coating concentration, serum dilution, incubation duration, wash cycle, and cut-off definition should be confirmed against the reagent package insert or laboratory validation data (22, 35).

3. Positive Criteria.

A sample is considered positive if its OD value exceeds twice that of the negative control or a predefined threshold based on the standard curve.

4. Notes.

Protein coating concentration must be carefully controlled to avoid reduced sensitivity. For recombinant full-length p53 protein-based ELISA, antigen aliquots should be prepared according to the manufacturer’s instructions or validated laboratory SOPs, stored at the recommended temperature, protected from repeated freeze-thaw cycles, and verified with positive and negative controls before clinical use.

#### 3.3.2 Western blot

1 Principle.

Western blot separates proteins via electrophoresis and detects target proteins or antibodies using specific antibodies.

2. Materials and Reagents.

The materials and reagents for Western blot include denatured recombinant p53 protein compatible with SDS-PAGE, PVDF or nitrocellulose transfer membrane, 5% skim milk or 3% BSA as the blocking buffer, patient serum or plasma diluted at 1:100 as the primary antibody source, HRP-conjugated anti-human IgG antibody as the secondary antibody, and chemiluminescent substrate (ECL) as the detection reagent.

3. Experimental Procedure.

The experimental procedure begins by separating recombinant p53 protein using SDS-PAGE, followed by transfer of separated proteins to a PVDF membrane at 100 V for 1 hour. The membrane is blocked with blocking buffer at room temperature for 1 hour, then incubated with diluted patient serum or plasma overnight at 4 C. After secondary antibody incubation with HRP-conjugated anti-human IgG antibody at room temperature for 1 hour, the signal is developed using an ECL substrate system and exposed to visualize specific bands.

4. Data Analysis.

Evaluate the specificity and intensity of bands. A positive result requires a clear band at the expected molecular weight of the target protein.

5. Notes.

The membrane should be blocked immediately after transfer to prevent protein degradation, and high-sensitivity chemiluminescent reagents are recommended to improve the signal-to-noise ratio.

#### 3.3.3 Protein microarray

1. Principle.

Multiple antigens are immobilized on a microarray chip, and antibody binding is detected using fluorescence labeling.

2. Materials and Reagents.

The materials and reagents include an antigen chip containing p53 and other relevant proteins, deionized water with Tween-20 as wash buffer, fluorescent dye-conjugated anti-human IgG antibody as the fluorescently labeled secondary antibody, and a laser scanner for detecting fluorescence signals (21, 36). This format is best interpreted as a multiplex research platform rather than a stand-alone clinical assay.

3. Experimental Procedure.

The experimental procedure begins with sample incubation, in which diluted patient serum is applied to the chip and incubated under validated conditions (21, 36). Unbound antibodies are then removed by washing with wash buffer, followed by incubation with fluorescently labeled anti-human IgG antibody in the dark. Signal detection is performed by scanning the chip with a laser scanner and recording fluorescence intensity (21, 36).

4. Data Analysis.

Signals are quantified and normalized using microarray analysis software, and a positive result is defined as fluorescence signal intensity exceeding twice that of the negative control.

5. Notes.

The chip should be handled under light-protected conditions to prevent fluorescence quenching and stored in a dry, low-humidity environment to avoid contamination.

#### 3.3.4 Electrochemical sensor

1. Principle.

Electrochemical sensors detect changes in current or voltage resulting from antigen-antibody binding using electrochemical techniques (34, 35). Although promising for miniaturized and rapid testing, these platforms remain in the exploratory stage for p53-AAb detection and require further analytical validation.

2. Materials and Reagents.

The materials and reagents include a glassy carbon or gold electrode, p53 protein immobilized on the electrode surface as the antigen, PBS or another compatible electrolyte solution as the detection solution, and diluted patient serum as the sample (34, 35).

3. Experimental Procedure.

Electrode modification: chemically immobilize p53 protein on the electrode surface (34). Sample reaction: apply diluted patient serum to the modified electrode and incubate under validated conditions. Electrochemical detection: record current changes using cyclic voltammetry or differential pulse voltammetry (34, 35).

4. Data Analysis.

Changes in current signal are proportional to antibody concentration. Calculate target antibody concentration using a standard curve.

5. Notes.

Uniform modification of the electrode surface should be ensured to prevent signal instability, and operations should be performed in a clean environment free from electromagnetic interference.

## 4 Clinical applications

### 4.1 Application of p53 autoantibodies in early cancer screening

The p53 gene is one of the most extensively studied genes (1, 2). p53-AAb can be used as a supplementary serological marker for early diagnosis and screening of several tumors (3, 21). In some cohorts, p53-AAb has been detected before overt clinical diagnosis or during early lesions, supporting its potential value as an early immune-response biomarker (7, 8). p53-AAb has been reported in breast, lung, and colorectal cancer (5, 9, 20). It has also been reported in gastric and esophageal cancer, where interpretation often overlaps with prognosis and multi-marker risk assessment (11, 12, 21) (**Table 2**). Because sensitivity is limited, it should be interpreted together with imaging, endoscopy, established tumor markers, and clinical risk factors rather than used as a stand-alone screening test (20, 24, 29).

**Table 2:** Summary of clinical evidence and interpretation of p53-AAb across selected cancer types.

Cancer type	Main evidence cited	Clinical role	Diagnostic interpretation	Recommendation implication
Gastric cancer	(11, 15, 27, 28)	Auxiliary screening/risk stratification; prognostic signal	High specificity, limited sensitivity; use with endoscopy/biopsy and CEA/CA72-4	Adjunctive use only
Colorectal cancer	(9, 10, 24)	High-risk screening adjunct and postoperative baseline monitoring	Improves interpretation when combined with CEA/CA19-9/FIT and imaging	Adjunctive use with defined thresholds
Breast cancer	(5, 7, 25, 26)	Supplement to imaging or autoantibody panels	Adjunctive serological information; interpret with mammography, clinical risk, and pathology	Do not replace mammography
Lung cancer	(8, 13, 20, 29)	Auxiliary marker for high-risk assessment	Auxiliary marker with limited sensitivity; not suitable as stand-alone screening	Use with imaging and risk models
Esophageal cancer	(12, 18, 31, 32)	Auxiliary screening of high-risk subjects; multi-marker diagnosis; prognostic support	Limited sensitivity for a single marker; performance improves in multi-autoantibody panels; interpret with endoscopy/biopsy and imaging	Adjunctive use in high-risk assessment and follow-up; not a stand-alone test
Oral cancer, especially OSCC	(37–41)	Adjunctive screening, prognostic assessment, and follow-up support	Evidence supports a subset of OSCC with detectable p53-AAb in serum or saliva; association with p53 pathway alteration, nodal involvement, and poorer survival has been reported	Use only as an adjunct to oral examination, biopsy, imaging, and histopathology; do not use as a stand-alone diagnostic test

#### 4.1.1 Gastric cancer

Previous studies have indicated that p53-AAb positivity in gastric cancer is associated with poorer survival outcomes and a more aggressive phenotype, highlighting its potential as an adverse prognostic biomarker (11, 27). Clinical studies also report that serum p53 antibody may help identify early gastric cancer or high-risk disease when interpreted with conventional tumor markers and endoscopic findings (15, 28). Therefore, in individuals with endoscopically suspicious but not yet confirmed lesions, p53-AAb may be used in combination with endoscopy/biopsy to guide stratified decision-making (15, 28). During postoperative follow-up or systemic therapy, dynamic monitoring of p53-AAb is exploratory; decreasing or clearing titers may be reassuring only when baseline positivity is documented, whereas re-positivity should prompt integrated evaluation rather than automatic diagnosis of recurrence (21, 27). These evaluations should be conducted in conjunction with CEA and CA72–4 to improve clinical reliability (21, 28, 30).

#### 4.1.2 Colorectal cancer

Screening high-risk groups with p53-AAb may support earlier detection of colorectal neoplasia when used as an adjunctive serological test (9, 24). Current clinical tumor markers such as CEA and CA19–9 are broad-spectrum and lack sufficient specificity when used alone (10). Systematic reviews and meta-analyses have shown that p53-AAb demonstrates high specificity but limited sensitivity in colorectal cancer (9, 24). When combined with CEA and CA19-9, p53-AAb can improve diagnostic interpretation and may help establish an individualized preoperative baseline (10). Thus, p53-AAb is best considered a useful serological adjunct for high-risk populations, such as those with family history, previous adenomas, or inflammatory bowel disease, rather than a replacement for colonoscopy, FIT, or imaging (9, 10, 24).

#### 4.1.3 Breast cancer

Studies indicate that p53-AAb can be detected in a subset of patients with breast cancer and may provide complementary serological information, particularly when interpreted together with imaging findings and established clinical risk factors (5, 7, 16). A diagnostic-test-accuracy review and meta-analysis of serum autoantibodies for breast cancer suggests that autoantibody approaches may contribute to early detection panels, but individual markers have limited sensitivity (25). Therefore, p53-AAb should be considered an adjunctive marker rather than a replacement for mammography or pathological diagnosis (25, 26).

#### 4.1.4 Lung cancer

When used in conjunction with imaging techniques and risk assessment, p53-AAb may contribute to early diagnostic assessment for lung cancer (13, 20). Published lung-cancer autoantibody studies and meta-analyses support its use as an auxiliary marker with relatively high specificity but limited sensitivity (17, 20, 29). Therefore, p53-AAb should not be used as a stand-alone screening test and should be interpreted with low-dose CT, smoking history, age, and other risk factors (20, 29).

#### 4.1.5 Oral cancer, especially oral squamous cell carcinoma

p53-AAb also has potential relevance in oral cancer, particularly oral squamous cell carcinoma (OSCC). In patients with oral lesions, circulating p53 antibodies were associated with p53 and HSP70 overexpression in the corresponding lesions, and seropositive patients had poorer disease-free survival than seronegative patients (37). In a cohort of 113 patients with primary oral SCC, serum p53-AAb positivity was 16%; positivity was associated with secondary cervical lymph-node metastasis, and p53-AAb-positive patients had lower 5-year survival than seronegative patients (56.2% vs. 80.7%) (38).

The biomarker may also support non-invasive detection approaches. p53 antibodies were detected in serum and saliva only in patients whose oral tumors showed p53 overexpression, suggesting that p53-AAb can identify a subset of oral tumors with p53 pathway alterations (39). More recently, salivary p53-AAb positivity was higher in patients with OSCC than in controls and was associated with lymph-node involvement, while an automated capillary immunoassay showed analytical performance comparable to a conventional ELISA platform (40, 41). These findings support p53-AAb as a possible adjunct for OSCC detection, risk stratification, and follow-up, but its limited sensitivity and heterogeneous evidence mean that it should not replace oral examination, biopsy, imaging, or histopathological diagnosis.

### 4.2 Advantages of p53 autoantibodies in early cancer screening

Serum autoantibodies offer several advantages over other serum proteins in cancer diagnosis: they may appear during early immune recognition of tumor-associated antigens (3, 21); they are amplified through humoral immune responses and can be detectable against high serum-protein background (20, 21); and they can be measured using conventional ELISA or EIA techniques with acceptable reproducibility when preanalytical and analytical procedures are standardized (13, 22, 35).

Compared with circulating tumor antigens, tumor-associated autoantibodies may offer high specificity and biological stability, but their sensitivity is usually limited and varies across cancer type, stage, antigen panel, and assay platform (20, 21, 24, 25).

Therefore, tumor autoantibody detection is best positioned as a complementary biomarker strategy in cancer screening and risk stratification rather than as a stand-alone diagnostic replacement (20, 21, 29).

Overall, p53-AAb testing may be incorporated as an adjunctive component of comprehensive screening strategies for gastric, colorectal, breast, lung, and esophageal cancers when interpreted with established clinical pathways and other biomarkers.

### 4.3 p53 autoantibodies in cancer prognosis assessment

Elevated p53-AAb levels are associated with aggressiveness and poor prognosis in certain cancers, particularly gastric and esophageal cancer (11, 27, 31). Studies of esophageal squamous cell carcinoma indicate that p53-related autoantibody panels may support screening of high-risk subjects and may carry prognostic information (18, 31, 32). Dynamic monitoring of p53-AAb levels may reflect treatment efficacy and recurrence risk in selected patients, but evidence remains heterogeneous and should be interpreted with imaging, pathology, and standard tumor markers (21, 27).

In long-term cancer management and follow-up, p53-AAb testing may be considered as an adjunctive marker, particularly when a pre-treatment positive baseline is available.

### 4.4 p53 autoantibodies in monitoring treatment response

A significant decrease in p53-AAb levels after treatment may indicate favorable treatment outcomes when the patient was positive before treatment (21, 27). Persistently high or re-emergent levels may suggest residual disease or recurrence risk in selected patients, but this signal is not specific enough for independent clinical decision-making (19, 21, 31). Therefore, treatment-response monitoring should be interpreted together with imaging, pathology, and standard tumor markers (20, 21).

Dynamic p53-AAb detection may be used as a supplementary, non-stand-alone tool for treatment response evaluation when baseline positivity and assay consistency are documented.

## 5 Challenges and future perspectives

### 5.1 Current challenges

1. Sensitivity and Specificity.

Current ELISA-based p53-AAb assays show limited sensitivity, which restricts stand-alone use in cancer screening (9, 20, 24). Combining multiple tumor-associated autoantibodies or integrating autoantibodies with conventional biomarkers is recommended to improve performance (20, 21, 29, 32).

2. Limited Large-Scale Prospective Data.

Clinical validation of p53-AAb is uneven across cancer types. Lung and colorectal cancer have systematic-review or meta-analytic evidence supporting adjunctive diagnostic interpretation (20, 24, 29). Gastric and esophageal cancer evidence highlights prognostic and multi-marker value (11, 21, 31). Breast cancer evidence supports possible early-detection panels but not replacement of standard imaging or pathology (25, 26). More prospective, multi-center studies are needed for other cancer types.

3. Lack of Standardization.

Unified clinical pathways, cut-off values, reference materials, and quality-control procedures have not yet been established for routine p53-AAb testing (21, 22, 35).

### 5.2 Future directions

1. Develop multi-marker diagnostic models combining p53-AAb with protein markers such as CEA and CA125 or with other tumor-associated autoantibodies to enhance early diagnostic accuracy (20, 21, 29, 32).
2. Utilize machine learning or deep learning algorithms to predict tumor type and progression risk in p53-AAb-positive patients, but require independent validation before clinical implementation (21, 32).
3. Conduct real-world, multi-center studies to further validate the clinical utility of p53-AAb, including preanalytical standardization, assay reproducibility, clinical cut-offs, and longitudinal monitoring value (21, 22, 35).

## 6 Summary

As a non-invasive and tumor-associated biomarker, p53-AAb shows potential in early cancer screening, auxiliary diagnosis, prognosis evaluation, and treatment monitoring (3, 20, 21). The standardized considerations summarized in this Review Article support cautious clinical translation while emphasizing that p53-AAb should be interpreted together with established diagnostic pathways, imaging, pathology, and conventional tumor markers (20, 21, 24, 29). With advancing technology and ongoing research, p53-AAb may become a useful component of precision cancer medicine when supported by validated multi-marker panels and prospective clinical evidence (21, 32).
